# Regular Contributions in South London

**Published:** 1922-12

**Authors:** R. J. Coles

**Affiliations:** (Appeal Secretary, King's College Hospital)


### REGULAR CONTRIBUTIONS IN SOUTH LONDON.

**By R. J. COLES (Appeal Secretary, King's College Hospital).**

IN the Report of the Voluntary Hospitals Commission appointed by the Minister of Health to consider the financial position of the voluntary hospitals, and to make recommendations as to any action which should be taken to assist them, the question of mass contributions or weekly contributions from workers was not overlooked. Indeed, the evidence both from the hospitals which could be looked upon as solvent, and those which were rapidly approaching a state of insolvency, convinced the Commission that if the voluntary hospitals are to continue their triple activities, they must rely not only on large subscriptions and donations, but upon the more moderate and regular contributions from all classes of the community.

This does not mean that the support of those who are in a position to give a substantial annual subscription or donation is no longer needed ; far from it, their support is needed as much as ever—much more the generous support of the whole community is needed if these great institutions are to continue and extend their work on behalf of suffering humanity. It is the willing support of the many, and not the generosity of the few, which will save our voluntary hospitals and maintain and extend their activities. This is comparable with the experience of recent years in the commercial world. Most men of business will admit that the day of reliance upon the generous patronage of a few clients has passed only to be superseded by regular patronage of the many, a much more healthy state of affairs. Thus, in hospital life of the future with an ever-increasing number of regular supporters, the inevitable losses caused by death and removal will be far less serious than in the old days when these institutions depended very largely upon the support of the few. This, surely, is a tremendous advantage.

The masses are not altogether to blame for not having given more generous support to the voluntary hospitals in the past. They have not been informed sufficiently of the work and needs of these great institutions. The fact that each individual from the cradle to the grave is tremendously indebted to hospitals has seldom, hitherto, been explained to them. Some will say, " How is such a gigantic task to be accomplished ? " Such contentions are not justifiable until the masses of workers in London have been approached. Is it possible to approach them ? It is possible, with the sympathy and co-operation of the employers.

### A Delusion of Londoners.

It has often been said that the system adopted by the workers in the provinces is not applicable to London, and cannot be adopted. This is not only a delusion, but an unjust reflection upon the masses of workers in the great metropolis. Experience has shown that with the co-operation of employers, by allowing a representative from the hospital, rather than anyone else, to attend at their place of business to give an address of, say, 15 minutes duration, to their employees upon the work and urgent needs of the hospital, in nearly every instance is secured the sympathy, the confidence and goodwill of the workers. The workers will then give their response in a practical manner by agreeing to give a definite sum of, at least, 2d. per week each. Their word is their bond, for as long as they are in employment they keep up their contributions, and many render additional assistance by influencing their colleagues to help the hospitals in a similar manner.

### First Results in South London.

In response to the Appeal made by King's College Hospital to the workers in the district which it serves, no less than 5,000 workers, men, women and girls, have come forward during the past year, to help, and are giving their regular support to that Hospital by contributing from 2d. to 3s. 6d. per week each. In some instances the employers have agreed to contribute a sum equal in amount to that given by their employees during the year. Thus it will be seen that the employees are leading the way, and in due course this splendid example is bound to influence other employees. In one parish where there is considerable unemployment no fewer than 480 men, women and girls have combined together and are contributing at least 2d. per week each to the funds of their Hospital. Some of these are also contributing at their place of employment. This shows that, although it may not always be possible to get into touch with the workers at their place of employment, there are other facilities for approaching them.

### A New Income of £13,000 a Year.

Amongst the 5,000 workers mentioned above, two cases are particularly significant ; the one, where a widow earning 28s. per week and having to bear the expense of daily travelling to and from her place of employment is contributing 1s. per week ; the other, a working man, married, earning about 50s. per week, contributes 6d. per week at his place of employment, and in addition sends regularly each week 3s. direct to the Hospital, thereby contributing 3s. 6d. per week. To the hospital in question, in addition to the regular support now being given by the workers, the patients and their friends are contributing something like £13,000 a year. It will thus be seen that if the workers are approached in the right spirit, and are made to feel that they should take an active interest in the voluntary hospitals, they will give regular weekly contributions and assist in other ways.

### A Practical Policy.

The Hospitals Saving Association has been established in order to obtain and co-ordinate the support of the workers and their employers. The first step, surely is to acquaint the employers and their employees of the object of the Association, and impress upon them that the salvation of the voluntary hospitals is in their hands. Secure their sympathy, their confidence and goodwill and the rest will be easy. This done, arrange for speakers who have had practical hospital administration and financial experience to address them. Encourage the employers and workers not only to give their support in the form of a weekly contribution, but to take an active interest by serving in Local or Group Committees for carrying out propaganda work.

### THE LONDON HOSPITAL AND THE ELECTION.

EVEN in ordinary times an institution like the London Hospital has to rebut many wild assertions made by imperfectly informed outsiders, but it is something new that any political body should seek to make capital at election time out of a voluntary hospital. The attempt has, however, been made. The Labour Party in Whitechapel lately circulated a leaflet alleging that the Borough Council, on which it had a majority, was arranging with the London Hospital for Finsen Light treatment " which," the leaflet declared, " hitherto only well-to-do people were able to afford." The London Hospital, of course, replied that it had been " treating the poorest citizens in the Finsen Light department for the last 20 years." Was it possible that Whitechapel was really ignorant of this ? However, this strange attempt to secure support at the Municipal and Parliamentary elections was soon checked. It is the more remarkable since Labour, more than any other party, is jealous at the barest breath, in times of unrest, that a voluntary hospital " takes sides." The above is worth recording as perhaps the strangest *canard* of this election.

